# Preclinical Metabolism, Pharmacokinetics and *In Vivo* Analysis of New Blood-Brain-Barrier Penetrant Fingolimod Analogues: FTY720-C2 and FTY720-Mitoxy

**DOI:** 10.1371/journal.pone.0162162

**Published:** 2016-09-09

**Authors:** Julius O. Enoru, Barbara Yang, Sesha Krishnamachari, Ernesto Villanueva, William DeMaio, Adiba Watanyar, Ramesh Chinnasamy, Jeffrey B. Arterburn, Ruth G. Perez

**Affiliations:** 1 In Vitro and Molecular Metabolism Laboratory, Ricerca Biosciences LLC, Concord, Ohio, United States of America; 2 Department of Biomedical Sciences, Graduate School of Biomedical Sciences, Center of Emphasis in Neurosciences, Paul L. Foster School of Medicine, Texas Tech University Health Sciences Center El Paso, El Paso, Texas, United States of America; 3 Department of Chemistry and Biochemistry, New Mexico State University, Las Cruces, New Mexico, United States of America; Northeastern University, UNITED STATES

## Abstract

Parkinson’s disease (PD) is a neurodegenerative aging disorder in which postmortem PD brain exhibits neuroinflammation, as well as synucleinopathy-associated protein phosphatase 2A (PP2A) enzymatic activity loss. Based on our translational research, we began evaluating the PD-repurposing-potential of an anti-inflammatory, neuroprotective, and PP2A stimulatory oral drug that is FDA-approved for multiple sclerosis, FTY720 (fingolimod, Gilenya®). We also designed two new FTY720 analogues, FTY720-C2 and FTY720-Mitoxy, with modifications that affect drug potency and mitochondrial localization, respectively. Herein, we describe the metabolic stability and metabolic profiling of FTY720-C2 and FTY720-Mitoxy in liver microsomes and hepatocytes. Using mouse, rat, dog, monkey, and human liver microsomes the intrinsic clearance of FTY720-C2 was 22.5, 79.5, 6.0, 20.2 and 18.3 μL/min/mg; and for FTY720-Mitoxy was 1.8, 7.8, 1.4, 135.0 and 17.5 μL/min/mg, respectively. In hepatocytes, both FTY720-C2 and FTY720-Mitoxy were metabolized from the octyl side chain, generating a series of carboxylic acids similar to the parent FTY720, but without phosphorylated metabolites. To assess absorption and distribution, we gave equivalent single intravenous (IV) or oral doses of FTY720-C2 or FTY720-Mitoxy to C57BL/6 mice, with two mice per time point evaluated. After IV delivery, both FTY720-C2 and FTY720-Mitoxy were rapidly detected in plasma and brain; and reached peak concentrations at the first sampling time points. After oral dosing, FTY720-C2 was present in plasma and brain, although FTY720-Mitoxy was not orally bioavailable. Brain-to-plasma ratio of both compounds increased time-dependently, suggesting a preferential partitioning to the brain. PP2A activity in mouse adrenal gland increased ~2-fold after FTY720-C2 or FTY720-Mitoxy, as compared to untreated controls. In summary, FTY720-C2 and FTY720-Mitoxy both (**i**) crossed the blood-brain-barrier; (**ii**) produced metabolites similar to FTY720, except without phosphorylated species that cause S1P_1_-mediated-immunosuppression; and (**iii**) stimulated in vivo PP2A activity, all of which encourage additional preclinical assessment.

## Introduction

Parkinson’s disease (PD) is a progressive multisystem neurodegenerative disorder in which the loss of nigral dopaminergic neurons leads to the characteristic motor symptoms of the disease [1]. Although PD is mainly sporadic and associated with aging, α-synuclein (αSyn), a chaperone-like protein, is highly implicated in PD by gene mutations and multiplications [2–8] and by αSyn accumulation in the pathological hallmarks of PD, the Lewy bodies [9], which are found in most PD brains. Although αSyn contributes to pathology, it also performs normal cellular functions [10–12] such as the ability to attenuate the activity of tyrosine hydroxylase (TH), the rate-limiting enzyme in dopamine biosynthesis [13]. A functional interaction also exists between αSyn and the PP2A catalytic subunit that stimulates PP2A activity [14–17]. Thus, aberrantly high levels of soluble αSyn or the loss of soluble αSyn, when it becomes sequestered in Lewy bodies, both can impair the normal regulation of TH and PP2A [18–20]. The relationship between αSyn and PP2A led us and others to consider PP2A as a potential therapeutic target for PD [15, 20, 21]. Moreover, we were the first to show that αSyn localizes to mitochondria [13], as corroborated by others [22–24], and it is known that αSyn aggregation can impair mitochondrial function [25, 26]. In addition, PD brain exhibits widespread mitochondrial damage [27]. Thus, therapies that enhance mitochondrial function may be particularly promising for treating PD and related synucleinopathies. These concepts led us to investigate the potential therapeutic application of FTY720 for PD, and also to develop new FTY720-based compounds that can enhance FTY720 efficacy or specifically target the drug to regions enriched in mitochondria [17].

FTY720, also called fingolimod or Gilenya, is a synthetic orally bioavailable sphingosine-1-phosphate receptor modulator that can stimulate PP2A activity [17, 28, 29]. The drug is FDA approved for treating multiple sclerosis (MS), and has provided significant benefits to MS patients worldwide [30, 31]. Furthermore, numerous studies [32–34], including our recent publication [17], demonstrate anti-inflammatory and neuroprotective effects rendered by FTY720 in vitro and in vivo. In our recent study [17], we described the effects of the FTY720 parent compound and our two newly synthesized FTY720-based compounds, a ceramide based analog FTY720-C2 and a β-triphenylphosphoniumpropanamide (Mitoxy) derivative FTY720-Mitoxy. With regard to PP2A activity, all three FTY720s have the ability to stimulate PP2A catalytic subunit activity, as well as increase brain-derived neurotrophic factor (BDNF) expression, and suppress TNF-α-toxicity in dopaminergic neuronal cells [17]. Beneficial effects of FTY720 on TNF-α have also been shown in microglial cells [35]. As seen in our recent study, FTY720-C2 tends to be more potent at stimulating PP2A activity than FTY720 or FTY720-Mitoxy at lower doses, and both new compounds significantly increase BDNF expression in neuronal cells [17] similar to the actions of FTY720 in various animal models [34, 36–38]. Having noted these promising findings, we chose to assess the drug metabolism and pharmacokinetic (DMPK) properties of FTY720-C2 and FTY720-Mitoxy as described herein.

The full absorption, distribution, metabolism, and excretion (ADME) data on FTY720 (fingolimod) are well described for humans [39–41]. FTY720 blood concentrations slowly reach maxima at ~ 12 to 24 hr after oral dosing and follow a slow decline with elimination half-life of about 7 days [42]. FTY720 is highly metabolized in human, and three biotransformation pathways have been reported, that include the reversible formation of FTY720 phosphate by sphingosine kinase; acylations with endogenous fatty acids at the amino group of FTY720; ω-hydroxylation at the octyl chain by CYP4F to form the octyl-alcohol metabolite M12 (not observed in vivo), and further oxidation to a carboxylic acid metabolite M1 (octanoic acid metabolite), followed by subsequent β-oxidation to generate a series of carboxylic acids (M2-M4) that are excreted in the urine [40, 41].

In our present study we describe the metabolic stability of FTY720-C2 and FTY720-Mitoxy in liver microsomes from 5 species as well as the drugs’ metabolic profiles in rat and human hepatocytes. We also show the pharmacokinetics determined after oral or intravenous dosing of mice with FTY720-C2 and FTY720-Mitoxy. In addition, we demonstrate the ability of these novel FTY720-analogues to stimulate PP2A activity in the adrenal glands of mice treated with single doses of FTY720-C2 and FTY720-Mitoxy. In summary, our novel FTY720-derived compounds show promising features: bioavailability in brain, metabolite profiles similar to that of marketed FTY720, and an ability of FTY720-C2 and FTY720-Mitoxy to stimulate PP2A activity in vivo.

## Materials and Methods

### Materials/Animals

Midazolam, diclofenac, glucose-6-phosphate dehydrogenase, glucose 6 phosphate, magnesium chloride, and testosterone (Sigma-Aldrich, St. Louis, MO); HPLC grade water, methanol, formic acid, and acetonitrile (Fisher Scientific, Pittsburgh, PA); cell free HepatoZYME-SFM (1x) medium (Gibco Life Technology, Grand Island, NY); C17 Sphingosine (Santa Cruz Biotechnology, Dallas, TX); Fingolimod hydrochloride salt (FTY720) (LC laboratories, Woburn, MA); the new compounds FTY720-C2 [*N*-(1-hydroxy-2-(hydroxymethyl)-4-(4-octylphenyl)butan-2-yl)acetamide] (MW 349.51 g/mol) and FTY720-Mitoxy [*N*-(1-hydroxy-2-(hydroxymethyl)-4-(4-octylphenyl)butan-2-yl)-3’-triphenylphosphoniumpropanamide] (MW 704.72 g/mol) were synthesized in Dr. Jeffery Arterburn’s laboratory (New Mexico State University, Las Cruces, NM) [17]. All reagents used were of analytical grade. Liver microsomes from mouse (CD-1, male, Lot number: 1110071 and 1310211), rat (Sprague-Dawley, male, Lot number: 1310030), dog (Beagle, male, Lot number: 1110044), monkey (Cynomolgus, male, Lot number: 1010321) and human (mixed gender, Lot number: 0910312 and 1216223) were purchased from Xenotech LLC (Lenexa, KS). Cryopreserved hepatocytes from rat (Sprague Dawley) and human were purchased from BioreclamationIVT (Baltimore, MD). Male C57BL/6 mice used for PK studies were obtained from Charles River Laboratories (Portage, MI), acclimated for no less than 5 days before use in experiments. Mice that were experimentally naïve prior to the study were randomly assigned to treatment groups. Animal studies were conducted following ethical treatment of animals according to NIH Animal Care Guidelines on protocols approved by the Institutional Animal Care and Use Committee of Ricerca Biosciences LLC (Protocol # 031416). Mice were euthanized by CO_2_ inhalation until unresponsive, followed by decapitation.

### Methods

#### Incubation with liver microsomes

Liver microsomal incubations contained 0.1mg/mL microsomal proteins and 1 μM of FTY720-C2 or FTY720-Mitoxy in 100 mM potassium phosphate buffer (pH 7.4) ± an NADPH regenerating system (3.5 mM glucose 6-phosphate, 1.3 mM NADP^+^, and 0.4 units/mL glucose-6-phosphate dehydrogenase). Following incubations, reactions were terminated for analysis after 0, 10, 20, 30, and 60 min by the addition of acetonitrile containing C17 sphingosine (300 ng/mL) as the internal standard. Samples were vigorously mixed and centrifuged at 3,400 rpm for 10 min to pellet precipitated proteins. An aliquot of each supernatant was used for LC/MS analysis, for determination of the remaining FTY720-C2 and FTY720-Mitoxy. Similar incubations were performed using a cocktail solution containing 0.2 μM midazolam and 1 μM diclofenac to determine the metabolic activity of the microsomes as an additional control.

#### In vitro intrinsic clearance determination in liver microsomes

Samples resulting from liver microsomal incubations were analyzed by LC-MS/MS. Aliquots of the supernatants were injected onto an X-Bridge C18 column (2.1 × 50 mm, 3.5-μm particles) (Waters, Milford, MA), preceded by a SecurityGuard C18 guard column (4.0 × 2.0 mm; Phenomenex Inc.). Separations were accomplished with a gradient of 0.1% acetic acid in water (v/v) versus 0.1% acetic acid in acetonitrile: methanol (75:25, v/v) at a rate of 0.8 mL/min. The eluents were further applied into an equipped API 4000 QTrap mass spectrometer (AB Sciex, Foster City, CA) with electrospray ionization (ESI) interface by two coupled LC-10 AT pumps. Peak area ratios of FTY720-C2 or FTY720-Mitoxy against the C17 sphingosine internal standard were used to determine the amount of parent remaining compared to that at 0 min. The percent of FTY720-C2 or FTY720-Mitoxy remaining was calculated by dividing the peak area ratio obtained at each time point by that obtained at 0 min. The in vitro intrinsic clearance (CL_int_) was calculated by linear regression of the log percent of compound remaining versus time plots using Microsoft Excel, two independent experiments (n = 2) were performed for each compound and species. Then, the mean and standard deviation (SD), of two values for each condition, were calculated using Microsoft Excel with the “AVERAGE” and “STDEV” functions, respectively.

#### Incubation with rat and human hepatocytes

Cryopreserved hepatocytes were grown in culture and incubations were performed to generate samples to measure the formation of FTY720-C2 and FTY720-Mitoxy metabolites. Incubations of 1 mL medium containing 1 million cells and 1 μM of FTY720-C2 and FTY720-Mitoxy were conducted at 37° C in a 5% CO_2_ incubator for 0, 1 and 2 hr, with gentle shaking. Positive control incubations used a cocktail solution containing 10 μM testosterone and 10 μM diclofenac performed for 0, 1 and 2 hr, under similar conditions to confirm the metabolic activity of the hepatocytes. The reactions were terminated by adding 2 mL acetonitrile containing 0.02% formic acid, and thoroughly mixing with gentle vortexing. Samples were transferred into 6 mL vials and stored in the freezer (-70°C) overnight for protein precipitation. Following overnight precipitations, samples were centrifuged at 3,000 rpm for 10 min to pellet proteins. Supernatants were transferred into clean 6 mL vials and evaporated to dryness using a TurboVap concentrator system. Samples were reconstituted using 600 μL 20% acetonitrile in water, briefly vortexed to resuspend, then aliquoted for LC-MS/MS analysis. Controls incubations also included “No drug control” and “no cell control” samples treated in a similar manner as test article samples.

#### Metabolite profiling and identification in hepatocytes

Metabolite profiling and identification experiments were conducted by LC-MS/MS, composed of an HPLC system consisting of an Agilent Technologies 1200 Series Model G1322A Degasser, Agilent Technologies 1200 Series Model G1312A Binary Pump, Shimadzu HTc autosampler, and Valco Model E60 Flow Diversion Valve, and coupled to an API 4000 mass spectrometer. Separations were accomplished on a Gemini C18 column (150 × 2.0 mm, 5-μm particles; Phenomenex Inc., Torrance, CA), preceded by a SecurityGuard C18 guard column (4.0 × 2.0 mm; Phenomenex Inc.). A two-component mobile phase, consisted of a linear gradient of water containing 0.5% formic acid (v/v) and methanol:acetonitrile (1:1, v/v) containing 0.5% formic acid (v/v), was pumped at 0.3 mL/min. The first 4 min of flow was diverted away from the mass spectrometer. Analysis of the positive control samples was conducted with an HPLC system consisting of two Shimadzu LC-10ATvp pumps and a LEAP Pal autosampler. Separations were accomplished on a Luna C8(2) column (50 × 2.0 mm, 3-μm particles; Phenomenex Inc., Torrance, CA), preceded by a SecurityGuard C18 column (4.0 × 2.0 mm; Phenomenex Inc.). Separation was accomplished with a gradient of water containing 0.2% formic acid and 0.012% NH_4_OH (v/v) versus acetonitrile at an initial rate of 0.7 mL/min. An API 4000 QTrap mass spectrometer (AB Sciex, Foster City, CA) was used for analysis of positive control samples. Each mass spectrometer used was equipped with an electrospray ionization source and operated in the positive ionization mode. Mass spectrometer settings for metabolite profiling are summarized in Table 1. Metabolite identification experiments were performed by recording full scan mass spectral data (Q1MS) for all samples, and selected samples were analyzed to obtain product ion spectra (MS/MS) of test article metabolites identified in the full scan mass spectral data.

**Table 1:** Mass Spectrometer Settings Used for Metabolite Profiling of FTY720-C2 and FTY720-Mitoxy.

Parameters	Value
Ion Spray Voltage (V)	5000
Curtain Gas (psi)	30
Temperature (°C)	500
Ion Source Gas 1 (psi)	60
Ion Source Gas 2 (psi)	60
Interface Heater	on
Declustering Potential (V)	65
Entrance Potential (V)	10
Collision Energy (for MS/MS)	22 to 27 eV

#### Oral and intravenous dosing of FTY720-C2 and FTY720-mitoxy in male C57BL/6 mice

The objectives of this study were to evaluate the pharmacokinetic profiles of FTY720-C2 and FTY720-Mitoxy following single oral and intravenous (IV) dosing of male mice and to determine blood and tissue levels of both compounds at various postdose time points. Male C57BL/6 mice were 8.4 weeks of age and weighed 20.6–24.8 g on the day of dose administration. Each animal was identified by a unique number via an ear tag with a matching cage tag. Mice were administered (two mice per time point) a single equivalent molar dose of either FTY720-C2 (1 mg/kg) or FTY720-Mitoxy (2 mg/kg) by oral or intravenous administration. As the molecular weight of FTY720-Mitoxy (704.72 g/mol) is ~ two times greater than that of FTY720-C2 (349.51 g/mol), the dose level of FTY720-Mitoxy was double that of FTY720-C2. Details of the design showing group assignments, dose groups, dose levels, as well as blood collection and necropsy time points are shown in Table 2. At the appropriate time points after dosing and blood sample collection for bioanalysis, animals were euthanized by CO_2_ inhalation until unresponsive, followed by decapitation. Brains were rapidly harvested, rinsed with ice cold saline, blotted dry and bisected along the mid-line. The left and right hemispheres of the brain were weighed separately. Each was placed in separate tubes, immediately flash frozen in liquid nitrogen and stored at -70°C prior to evaluation. All samples were labeled with the test facility study number, animal identification number, dose group, date, collection interval, and tissue identity (left or right brain hemisphere). Adrenal glands from each mouse were also collected and flash frozen.

**Table 2:** Pharmacokinetic Study Design for FTY720-C2 and FTY720-Mitoxy.

Test article	Route of Administration	N^a^	Dose Level (mg/kg)	Dose Conc. (mg/mL)	Dose Volume (mL/kg)	Blood Collection and Necropsy^b^ Time Points (Postdose)
FTY720-C2	Oral	8	1	0.2	5	1, 8, 24, and 48 hr
FTY720-Mitoxy	Oral	8	2	0.4	5	1, 8, 24, and 48 hr
FTY720-C2	Intravenous	8	1	0.5	2	5 min, 8, 24, and 48 hr
FTY720-Mitoxy	Intravenous	8	2	1	2	5 min, 8, 24, and 48 hr

^a^Two animals per time point (n = 2).

^b^Mouse brain and adrenal glands were collected upon necropsy for pharmacokinetic study and protein phosphatase assay, respectively.

#### FTY720-C2 and FTY720-mitoxy concentration determination in mouse plasma and brain

FTY720-C2 and FTY720-Mitoxy concentrations in plasma and brain were determined by LC-MS/MS. Samples were first prepared by liquid-liquid extraction. To 50 μL of plasma or brain homogenate were added 50 μL of 0.1 M NaOH and 50 μL of internal standard solution (C17 sphingosine) in ethanol. Analytes were then extracted into 1 mL of methyl tert-butyl ether:dichloromethane mixture (75:25, v/v) by vortexing for 1 hr and centrifuged. Supernatants were evaporated to dryness under nitrogen. Residues were reconstituted in 150 μL of 50% methanol in water and spin filtered before applied onto XBridge C18 column (3.5-μm particle; Waters, Milford, MA) with a SecurityGuard C18 guard column (4.0 × 2.0 mm; Phenomenex Inc.). Separations were accomplished with a gradient of 0.1% acetic acid in water (v/v) versus 0.1% acetic acid in acetonitrile:methanol (75:25, v/v) at a rate of 0.5 mL/min. The eluents were further applied into an equipped API 4000 QTrap mass spectrometer (AB Sciex, Foster City, CA) with electrospray ionization (ESI) interface by two coupled LC-10 AT pumps, and monitored in multiple reaction monitoring (MRM) mode with precursor to product ion pairs for C17 sphingosine, FTY720-C2, and FTY720-Mitoxy as following: *m/z* 286.3 to 268.2, *m/z* 350.3 to 255.2, and *m/z* 624.4 to 289.2, respectively.

FTY720-C2 mean concentrations in plasma and brain were plotted as bar graphs with SD calculated from the two values per time point, while FTY720-Mitoxy mean concentrations in plasma and brain were plotted as scatter graphs with mean and SD calculated using Prism 6 (GraphPad Software Inc., San Diego, CA, USA)

#### Pharmacokinetic calculations

Pharmacokinetic analysis was conducted using WinNonlin Version 6.2 (Pharsight, Mountain View CA), operating as a validated software system. Non-compartmental analysis was conducted using a bolus intravenous administration model for IV dosing and an extravascular administration model for oral dosing. The peak plasma concentration, time to achieve peak plasma concentration, half-life, mean residence time, and area under the plasma or brain concentration-time curve (C_max_, T_max_, T_1/2_, and AUC) were calculated from mean animal plasma and brain concentrations. Nominal blood collection times were used for pharmacokinetic calculations.

#### Protein phosphatase assay

Adrenal glands from treated mice were homogenized in buffer containing 20 mM imidazole-HCl, 2 mM EDTA, 2 mM EGTA, plus protease inhibitors, at 4°C. The homogenates were centrifuged to remove particulates followed by free phosphate removal on MicrospinTM-G-25 columns (GE Healthcare). Aliquots of supernatants were incubated in 4-Nitrophenyl-phosphate (pNPP) buffer consisting of 50 mM Tris-HCl, pH 7.0, 0.1 mM CaCl_2_ with threonine-phosphopeptide (KRpTIRR) substrate. The reactions were run for 10 min at 30°C and stopped by placing samples on ice. Phosphate levels were determined by measuring absorbance at 650 nm using a Multiskan Spectrum plate reader (Thermo Scientific, Pittsburgh, PA) and were compared to freshly prepared standards as previously described, but using a malachite green solution prepared according to Fathi et al. [15, 16, 20, 43]. Samples from two mice per time point (n = 2) were assayed in triplicate to confirm technical fidelity.

#### Statistics

Descriptive statistics were performed using Microsoft Excel or Prism 6 (GraphPad Software Inc., San Diego, CA, USA). Analysis of variance (ANOVA) was performed using Prism 6, with significance set to p < 0.05. Data represent mean ± standard deviation (SD) for all experiments, except for PP2A assays, which show the standard error of the mean (SEM) as ANOVA was performed.

## Results

### Metabolic Stability in Liver Microsomes

Preliminary investigations to establish the metabolism of FTY720-C2 and FTY720-Mitoxy were conducted by determining the in vitro intrinsic clearance (CL_int_) of the two novel compounds in liver microsomes from five different species—mouse, rat, dog, monkey and human. The mean intrinsic clearance values for the two new compounds are summarized in Table 3. The intrinsic clearance of FTY720-C2 in mouse, rat, dog, monkey and human liver microsomes was 22.5, 79.5, 6.0, 20.2 and 18.3 μL/min/mg (average of two experiments), respectively. The in vitro intrinsic clearance of FTY720-C2 was determined to be low in dog; moderate in mouse, monkey and human; and high in rat. For FTY720-Mitoxy, the intrinsic clearance in mouse, rat, dog, monkey and human liver microsomes was 1.8, 7.8, 1.4, 135.0, and 17.5 μL/min/mg (average of two experiments), respectively, with the intrinsic clearance interpreted to be low in mouse, rat and dog; moderate in human; and high in monkey. Intrinsic clearance of FTY720, used as a control, was determined to be slow in microsomes of all five species (data not shown). After 60 minute incubations in liver microsomes, the percent of the remaining compound in mouse, rat, dog, monkey, and human liver microsomes was 29.5 ± 2.1%, 1.0 ± 0%, 69.9 ± 0.6%, 30.0 ± 0.3%, and 33.5 ± 4.5%, respectively for FTY720-C2; whereas it was 82.5 ± 16.3%, 65.5 ± 4.9%, 112.5 ± 5.0%, 1.4 ± 0.3%, and 35.5 ± 2.3%, respectively for FTY720-Mitoxy.

**Table 3:** Intrinsic Clearance in Liver Microsomes.

	FTY720-C2	FTY720-Mitoxy
Species	Cl_int_ (μL/min/mg)^a^	Cl_int_ (μL/min/mg)^a^
Mouse	22.5 ± 2.12	1.75 ± 1.34
Rat	79.5 ± 1.41	7.8 ± 1.98
Dog	6.0 ± 0.28	1.35 ± 1.48
Monkey	20.2 ± 0	135.3 ± 10.61
Human	18.3 ± 2.4	17.5 ± 1.70

Cl_int_—Intrinsic clearance.

^a^Values represent the mean ± SD of two determinations.

### Metabolite Profiles and Structures in Hepatocytes

The metabolite profiles of FTY720-C2 and FTY720-Mitoxy were determined by LC-MS/MS. Metabolites formed in significant quantities were further identified by MS/MS analysis as discussed below. Structural characterization of FTY720-C2 and FTY720-Mitoxy metabolites, observed in significant amounts, was conducted by LC-MS/MS. Mass spectral data of FTY720-C2, FTY720-Mitoxy, and their metabolites in rat and human hepatocytes are summarized in Tables 4 and 5, respectively, in order of increasing retention times. The *m/z* values indicated are expressed as nominal values obtained from addition of the integer atomic weights of the most abundant naturally occurring isotope of each element in the molecular formula. Structural characterization of the metabolites detected was tentatively assigned based upon comparison with the mass spectra and the proposed fragmentation schemes of the respective unchanged parent compounds. Minor metabolites for which no MS/MS data were obtainable were proposed based only on molecular weight data.

**Table 4:** Summary of the structural characterization by LC-MS/MS of FTY720-C2 Metabolites Identified in Rat and Human Hepatocytes.

Peak	Source^a^	LC-MS/MS t_R_ (min)^b^	MW	[M+H]^+^	Relevant Product Ions (*m/z*)
FTY720-C2 C2-carboxylic acid	R, H	20.5	295	296	278, 260, 254, 350, 201, 236, 218, 189, 173, 155, 149, 143, 117, 60
FTY720-C2 C4-carboxylic acid	R, H	24.1	323	324	306, 288. 282, 270, 264, 246, 229, 217, 211, 199, 177, 155, 117, 60
Dihydroxy FTY720-C2	h	24.5	381	382	Not available
FTY720-C2 C6-carboxylic acid	r	27.9	351	352	Not available
FTY720-C2 C8-carboxylic acid	R, H	31.6	379	380	362, 344, 338, 320, 285, 273, 267, 255, 237, 173, 155, 143, 105, 60
Hydroxy FTY720-C2	R, H	32.1	365	366	348, 330, 324, 306, 271, 259, 253, 241, 173, 155, 143, 60
FTY720-C2	R, H	39.2	349	350	332, 314, 308, 290, 255, 243, 229, 203, 143, 131, 117, 105, 71, 60, 57

^a^ R = Rat; hr = Human; lowercase letter indicates low abundance.

^b^ LC-MS/MS retention time obtained from Q1MS data file(s) with preference given to the respective human sample.

**Table 5:** Summary of the structural characterization by LC-MS/MS of FTY720-Mitoxy Metabolites Identified in Rat and Human Hepatocytes.

Peak	Source^a^	LC-MS/MS t_R_ (min)^b^	MW	[M]^+^	Relevant Product Ions (*m/z*)
FTY720-Mitoxy C4-carboxylic acid	r, h	25.7	598	598	Not available
FTY720-Mitoxy C6-carboxylic acid	R, H	27.6	626	626	608, 390, 334, 289, 262, 143, 72
Hydroxy FTY720-Mitoxy	r	28.8	640	640	Not available
FTY720-Mitoxy C8-carboxylic acid	R, H	29.7	654	654	390, 334, 289, 262
Hydroxy FTY720-Mitoxy	h	30.8	640	640	622, 390, 334, 289, 262
FTY720-Mitoxy	R, H	34.3	624	624	606, 390, 334, 289, 262, 143, 105, 72

^a^ R = Rat; hr = Human; lowercase letter indicates low abundance.

^b^ LC-MS/MS retention time obtained from Q1MS data file(s) with preference given to the respective human sample.

### FTY720-C2 and its Metabolites

**Fig 1:**
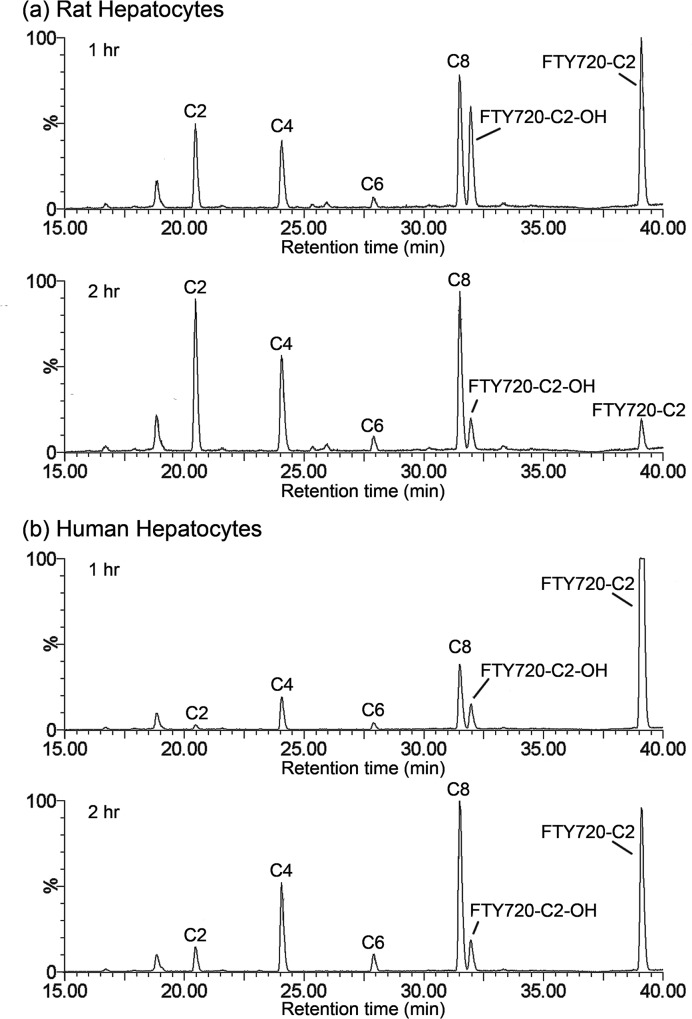
Representative LC/MRM Chromatogram of FTY720-C2 Metabolite Profile. Using 1 μM of FTY720-C2, we incubated rat (a) and human (b) hepatocytes to obtain the metabolite profile. To simplify presentation, the 0 min incubation time point, data are intentionally not shown. Legend: **C2**, FTY720-C2 C2-carboxylic acid; **C4**, FTY720-C2 C4-carboxylic acid; **C6**, FTY720-C2 C6-carboxylic acid; **C8**, FTY720-C2 C8-carboxylic acid; **FTY720-C2-OH**, hydroxy FTY720-C2.

**Fig 2:**
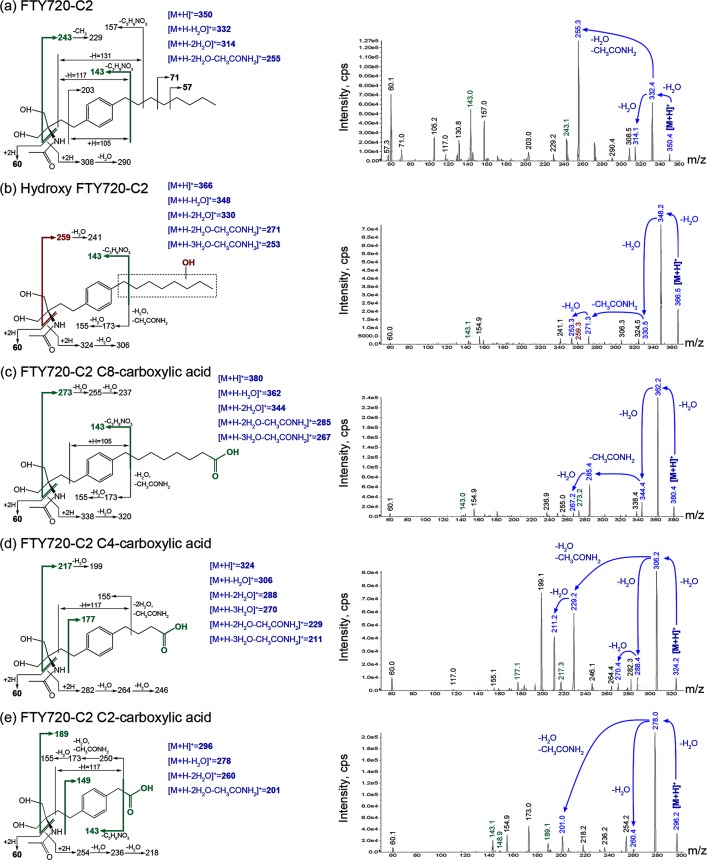
Proposed Fragmentation Schemes and Mass Spectra for FTY720-C2 and its Metabolites. Mass spectrometry of FTY720-C2 and its metabolites, detected as protonated molecular ions [M+H]^+^ are shown. Our rationale for identifying the structure of the metabolites is demonstrated using colored text and arrows, with **blue** product ions indicating losses of H_2_O and an *N*-acetyl group from [M+H]^+^, **red** product ions indicating hydroxylation, and **green** product ions associated with identified carboxylic acid modifications.

FTY720-C2 and its metabolites were assigned to representative peaks in the chromatogram (Fig 1) after identifying them by MS/MS (Fig 2). The mass spectrum ion intensity and proposed fragmentation scheme and products of [M+H]^+^ of FTY720-C2 are shown in Fig 2A. Neutral losses of water and the *N*-acetyl group generated product ions at *m/z* 332, 314, and 255, respectively (Fig 2A, blue text on the fragmentation scheme and mass spectrum). An unchanged *N*-acetyl moiety was indicated for FTY720-C2 and its metabolites by the product ion at *m/z* 60. Fragmentation of the octyl and ethylpropyl aliphatic chains generated the majority of the remaining observed product ions. The mass spectrum ion intensity and proposed fragmentation schemes of FTY720-C2 metabolites: hydroxy FTY720-C2, FTY720-C2 C8-carboxylic acid, FTY720-C2 C4 carboxylic acid, and FTY720-C2 C2-carboxylic acid are shown in Fig 2B–2E. For each FTY720-C2 metabolite, mass spectral data indicated the acetylamino moiety, phenyl ring and aliphatic carbon atoms closest to the phenyl ring as being unchanged. Protonated FTY720-C2 C8-, C4-, and C2-carboxylic acid metabolites generated no butyl or pentyl product ions at *m/z* 71 or 57, respectively, suggesting metabolism of the octyl side chain. Also, protonated FTY720-C2 C4- and C2-carboxylic acids metabolites generated fragments at *m/z* 177 and *m/z* 149, respectively, which indicated an elimination of a 2-carbon unit of the octyl side chain (Fig 2C and 2D, green text on the fragmentation schemes). Similarly, the *m/z* 243 product ion of FTY720-C2 shifting from *m/z* 273 to 217 and 189, for FTY720-C2 C8-, C4-, and C2-carboxylic acid metabolites, indicated a sequential and stepwise elimination of 2-carbon units (Fig 2C–2E, green text on the fragmentation schemes). In combination, the metabolites present in the chromatogram (Fig 1) were tentatively identified as FTY720-C2 C8-, C6-, C4- and C2-carboxylic acids. Hydroxylation of the octyl side chain including its benzyl carbon was proposed, partly based on the observation of the generation of multiple carboxylic acids from the octyl side chain. The fragment ion at *m/z* 243 of FTY720-C2 was shifted to *m/z* 259 (Fig 2B, red text on the fragmentation scheme), which correlated with a hydroxyl group replacing a hydrogen atom on the octyl side chain as fragment *m/z* 143 was seen for both FTY720-C2 and its metabolite (Fig 2A and 2B, green text on the fragmentation schemes). This common product ion at *m/z* 143 was observed in most of the metabolites, implying (**i**) that the C2 moiety remains unmodified, and (**ii**) that the metabolic transformation of FTY720-C2 occurred mainly by β-oxidation of the octyl side chain. Overall, our data suggest that FTY720-C2 was metabolized into a series of carboxylic acids and that hydroxylation might be the first step, as is known to occur for the parent FTY720 compound. However, unlike FTY720, no phosphorylation of FTY720-C2 was detectable.

### FTY720-Mitoxy and its Metabolites

**Fig 3:**
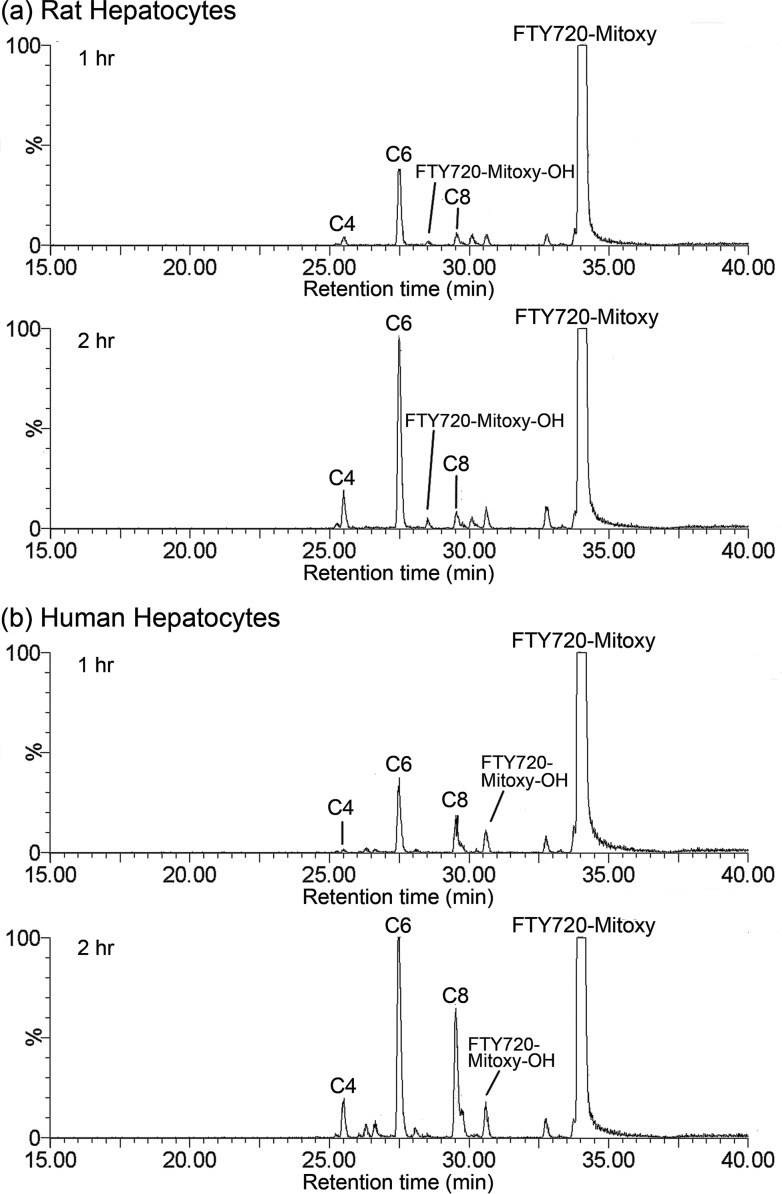
Representative LC/MRM Chromatogram of FTY720-Mitoxy Metabolite Profile. Using 1 μM of FTY720-Mitoxy, we incubated rat (a) and human (b) hepatocytes to obtain metabolite profiles. To simplify presentation, the 0 min incubation time point data are intentionally not shown. Legend: **C4**, FTY720-Mitoxy C4-carboxylic acid; **C6**, FTY720-Mitoxy C6-carboxylic acid; **C8**, FTY720-Mitoxy C8-carboxylic acid; **FTY720-Mitoxy-OH**, hydroxy FTY720-Mitoxy.

**Fig 4:**
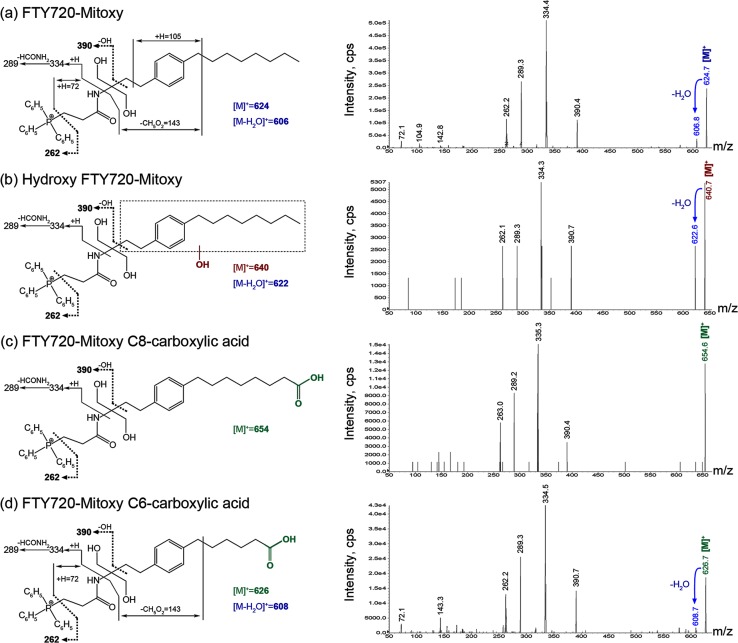
Proposed Fragmentation Scheme and Mass Spectrum for FTY720-Mitoxy and its Metabolites. Mass spectrometry of FTY720-Mitoxy and metabolites detected [M^+^] molecular ions. **Blue** text and arrows indicate losses of H_2_O from [M]^+^. The ion with a hydroxyl group is shown in **red**. Ions including a carboxylic acid are shown in **green**.

FTY720-Mitoxy and its metabolites were assigned to peaks in the chromatogram (Fig 3) after being identified by MS/MS (Fig 4). The positively charged phosphonium molecular ions [M]^+^ were detected in positive ionization mode. The proposed fragmentation scheme and products of the [M]^+^ mass spectrum of FTY720-Mitoxy and its metabolites are shown in Fig 4. The common product ion seen at *m/z* 390 and 262 among all MS spectra (Fig 4, black bold text and dashed line on the fragmentation schemes) indicated that the β-triphenylphosphoniumpropanamide group was unchanged for FTY720-Mitoxy and its metabolites; metabolism occurred toward the phenyl ring and the octyl side chain. A shift of *m/z* 624 to 640 suggested hydroxylation of FTY720-Mitoxy (Fig 4B, red text), and the site of hydroxylation was not unambiguously determined, but likely occurs at positions 4, 6, and/or 8 of the octyl moiety corresponding to the observed carboxylic acid metabolites. Although FTY720-Mitoxy and its proposed FTY720-Mitoxy C8- and C6-carboxylic acid metabolites did not generate product ions from the octyl side chain and corresponding metabolized octyl side chain, respectively, the tentative identification of the metabolites was consistent with molecular weight differences (Fig 4C and 4D, green text) versus the parent FTY720-Mitoxy and the expected oxidative metabolism of acyclic aliphatic compounds. Neither of new compounds had phosphorylated metabolites detected.

### Relative Percent Distribution and Time-Dependent Formation of Metabolites

Assuming that the LC/MS sensitivity of both test articles and their various metabolites are the same; Table 6 shows the relative abundance of metabolites generated in rat and human hepatocytes. In both rat and human cells, the oxidation of the octyl moiety in FTY720-C2 to carboxylic acids was the major route of metabolism, with FTY720-C2 C8-COOH being the most abundant metabolite, representing 37% and 39% in rat cells; and 49% and 52% in human cells, after incubation for 1 hr and 2 hr, respectively (Table 6). Oxidation of the octyl group was also the major route of metabolism for FTY720-Mitoxy. FTY720-Mitoxy C6-COOH, which results from oxidation of FTY720-Mitoxy C8-COOH, was the most abundant metabolite observed, constituting 84% and 79% in rat cells; and 59% and 55% in human cells after incubation for 1 hr and 2 hr, respectively (Table 6).

**Table 6:** Relative Percent Distribution of FTY702-C2 and FTY720-Mitoxy.

**FTY720-C2 Metabolites**	**[M+H]**^**+**^	**Percent Distribution (1 hr incubation)**		**Percent Distribution (2 hr incubation)**	
		**Rat**	**Human**	**Rat**	**Human**
FTY720-C2 C2-carboxylic acid	296	21.4	3.2	35.72	7.21
FTY720-C2 C4-carboxylic acid	324	9.7	23.7	12.88	25.57
FTY720-C2 C6-carboxylic acid	352	2.9	5.2	3.75	5.32
Hydroxy FTY720-C2	366	29.1	18.9	8.28	9.77
FTY720-C2 C8-carboxylic acid	380	37.0	49.0	39.37	52.13
**Total Percent Distribution**		100	100	100	100
**FTY720-Mitoxy Metabolites**	**[M]**^**+**^	**Percent Distribution (1 hr incubation)**		**Percent Distribution (2 hr incubation)**	
		**Rat**	**Human**	**Rat**	**Human**
FTY720-Mitoxy C4-carboxylic acid	598	8.4	2.3	14.0	9.5
FTY720-Mitoxy C6-carboxylic acid	626	83.6	58.6	79.3	54.8
Hydroxy FTY720-Mitoxy	640	3.5	19.1	3.2	8.3
FTY720-Mitoxy C8-carboxylic acid	654	4.4	20.0	3.4	27.3
**Total Percent Distribution**		100	100	100	100

### FTY720-C2 and FTY720-Mitoxy Pharmacokinetics in Plasma and Brain

**Fig 5:**
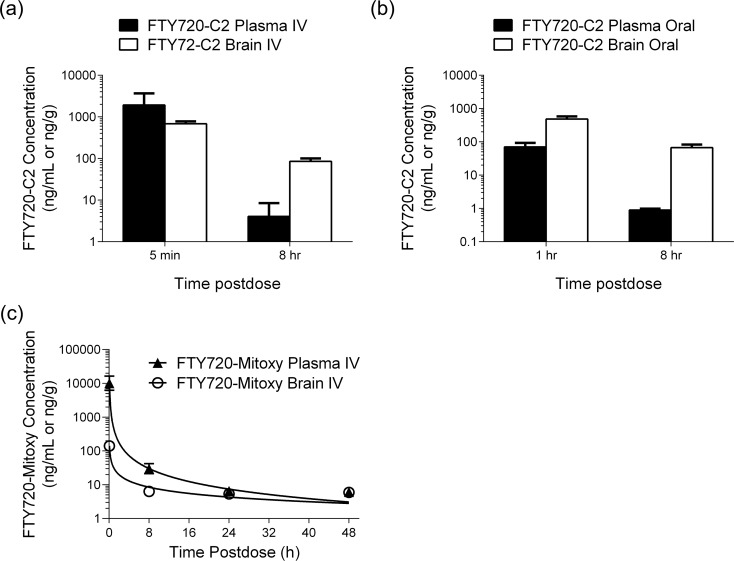
Plasma and Brain FTY720-C2 and FTY720-Mitoxy concentration profiles. Mean plasma and brain concentrations of FTY720-C2 after IV dosing (**a**) and oral dosing (**b**) (black bar = plasma; white bar = brain); and of FTY720-Mitoxy after IV dosing (**c**) (▲ = plasma; ○ = brain). Units are in ng/mL for plasma and ng/g for brain. Data represent the mean ± SD of two experiments for each time point. Error bars were calculated for all samples and are present on the graphs. However, for samples with little variability error bars do not extend beyond the edges of the symbols so thus, are not apparent.

Plasma and brain concentration versus time profiles of FTY720-C2 and FTY720-Mitoxy after IV or oral dosing of mice are shown in Fig 5. Following IV dosing, the highest FTY720-C2 concentrations in plasma and brain occurred at 5 min postdose, the first sampling time point (Fig 5A). Similarly, the highest concentrations for both tissues after oral administration of FTY720-C2 occurred at 1 hr (Fig 5B). However, FTY720-C2 levels were not measurable with either route at 24 hr and 48 hr postdose, the third and fourth sampling time points. As a result, pharmacokinetic parameters were not calculated. Nevertheless, our data demonstrated that FTY720-C2 became available in the brain rapidly, in 5 min with IV delivery and 1 hr with oral dosing. Notably, although FTY720-C2 dropped from the first sampling time point to the second, FTY720-C2 brain concentrations were much higher than those in plasma after both IV and oral dosing at 8 hr postdose (Fig 5A and 5B). For FTY720-Mitoxy, the pharmacokinetic parameters in plasma and brain were computed as data for all four different sampling time points obtained. For IV dosing, the T_max_ for both plasma and brain occurred at the same time (5 min), whereas the plasma and brain elimination half-life values were 0.94 and 1.77 hr, respectively. The FTY720-Mitoxy compound was not detected in plasma or brain following oraldosing, indicating the lack of bioavailability by this route of administration.

### In vivo PP2A Activity is stimulated by FTY720-C2 and FTY720-Mitoxy

**Fig 6:**
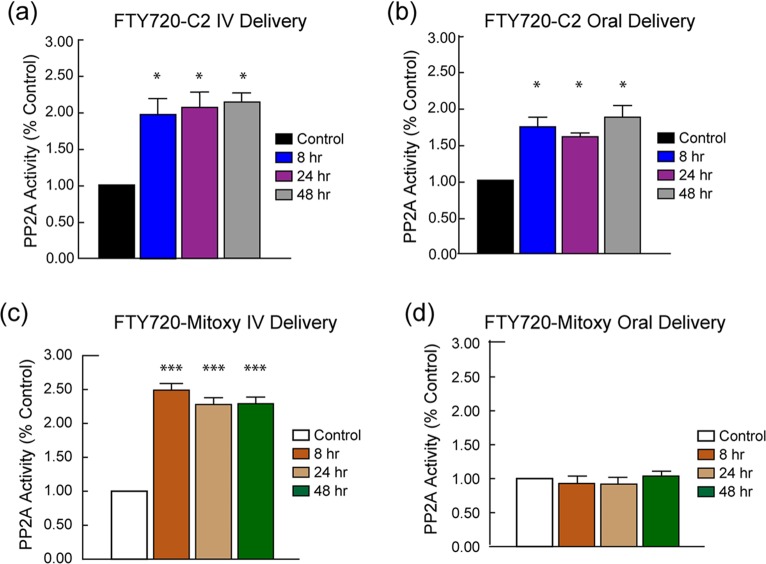
PP2A Activity in Mouse Adrenal Gland after Intravenous or Oral Dosing with FTY720-C2 or FTY720-Mitoxy. FTY720-C2 increased PP2A activity in adrenal glands at all time points following IV delivery (**a**) and oral delivery (**b**) as compared to untreated control mice. FTY720-Mitoxy increased PP2A activity in the adrenal gland at all time points after IV delivery (**c**). After oral delivery, FTY7220-Mitoxy was not absorbed and adrenal PP2A activity did not increase as compared to untreated controls (**d**). Two mice per time point were evaluated. Data represent mean ± SEM.

FTY720 is known to stimulate PP2A activity and we have previously demonstrated the in vitro activation of PP2A in response to our novel FTY720-based compounds [17], and here we assessed the effects of our novel FTY720 analogues on PP2A activity in treated mice. Adrenal glands from mice given single doses of FTY720-C2 or FTY720-Mitoxy by oral and IV delivery were used for this study. FTY720-C2 significantly increased PP2A activity by 2-fold and 1.6 to 1.8-fold throughout the sampling window after it was administered intravenously or orally, respectively (Fig 6A and 6B). PP2A activity was also significantly increased by FTY720-Mitoxy by 2.3 to 2.5-fold up to 48 hr postdose, but only after IV-dosing (Fig 6C). There was no stimulating effect on PP2A activity when FTY720-Mitoxy was orally administrated (Fig 6D), which correlates with the fact that FTY720-Mitoxy levels were not detected in mouse brain or plasma after oral delivery of the compound.

## Discussion

The in vitro intrinsic clearance (Cl_int_) of our novel FTY720 analogues from liver microsomes was found to be species-specific (Table 3). FTY720-C2 Cl_int_ was low in dog (6.0 μL/min/mg); moderate in mouse, monkey, and human (22.5, 20.2, and 18.3 μL/min/mg); and high in rat liver microsomes (79.5 μL/min/mg). For FTY720-Mitoxy, Cl_int_ was low in mouse, rat, and dog (1.75, 7.8, and 1.35 μL/min/mg); moderate in human (17.5 μL/min/mg); and high in monkey (135.3 μL/min/mg). In contrast, the intrinsic clearance of parent FTY720, run as an internal control in our studies, was low in all species (data not shown), which is also consistent with FTY720 Cl_int_ in previously reported liver microsome analyses [44]. These data suggest that the addition to FTY720 of the small C2 moiety or the larger triphenylphosphonium group made both novel compounds more labile in rat and monkey liver microsomes, respectively. Although species-dependent variability was noted for mouse, rat, dog and monkey, it is intriguing that human liver microsomes cleared FTY72-C2 and FTY720-Mitoxy at equivalent moderate rates of 18.3 and 17.5 μL/min/mg, respectively.

As done for FTY720 characterization [45], our in vitro FTY720-C2 and FTY720-Mitoxy metabolic profiles were also generated using rat and human hepatocytes. In both rat and human hepatocytes, we noted that the oxidation of the octyl chain of FTY720-C2 and FTY720-Mitoxy was the major metabolic pathway producing the formation of essential carboxylic acid metabolites (Figs 1 and 3), but at rates different than those seen for Cl_int_. The parent FTY20-C2 peak was drastically diminished by the 2 hr time point in rat hepatocytes (Fig 1A), but diminution was more gradual in human hepatocytes (Fig 1B). For parent FTY720-Mitoxy, there was no notable difference between rat and human hepatocytes after 2 hr incubation (Fig 3); when most FTY720-Mitoxy was still unchanged as compared to data for FTY720-C2 at this time point. These findings partly correlate with the in vitro metabolic stability data (Cl_int_) in which: rat liver microsomes showed higher clearance (~4-fold) than did human liver microsomes for FTY720-C2, but Cl_int_ in rat was lower (around half) that found in human microsomes for FTY720-Mitoxy. In terms of metabolites, hydroxylation of both FTY720-C2 and FTY720-Mitoxy were identified early in 1 hr incubations (Figs 1 and 3). As previously mentioned in the Introduction to this paper, one of the biotransformation pathways of FTY720 is ω-hydroxylation at the octyl chain being the first step, followed by further oxidation and subsequent β-oxidation to yield different length carboxylic acid metabolites [41]. Human hepatocytes generated similar levels of hydroxy FTY720-C2 and FTY720-Mitoxy (Figs 1B and 3B), but in rat hepatocytes, hydroxy FTY720-C2 was produced in larger amounts than was hydroxy FTY720-Mitoxy (Figs 1A and 3A); again, suggesting species-specific differences. Hydroxylation at the octyl chain is the initiating step for oxidative metabolism, so the data suggest that rat hepatocytes may have metabolized FTY720-C2 more readily than FTY720-Mitoxy. Also, the FTY720-C2 C2 carboxylic acid (C2), a further breakdown product of FTY720-C2 C4 carboxylic acid (C4), was detected at significant amounts in rat, but only in small amounts in human hepatocytes after 1 hr or 2 hr incubations (Fig 1), while the equivalent C2 metabolite was not observed for FY720-Mitoxy (Fig 3) or for FTY720, used as an internal control (data not shown). However, a FTY720 M4 metabolite (C2 carboxylic acid) has been detected in human urine as a minor product, for samples collected from 0 to 240 hr postdose [41]. Therefore, our data suggest that FTY720-C2 was metabolized further and faster than FTY720-Mitoxy or FTY720, at least at early time points (1 hr and 2 hr). Importantly, the modifications used to create FTY720-C2 and FTY720-Mitoxy, that is, the C2 moiety and the triphenylphosphonium group, remained unchanged although the octyl side chain was highly metabolized after 2 hr incubations with hepatocytes. This indicates that those modifications appear to be very stable. In addition, we never detected phosphorylated metabolites of FTY720-C2 or FTY720-Mitoxy despite the fact that phospho-FTY720 (FTY720-P) was identified when used as an internal control in the same rat and human hepatocytes (data not shown). It is noteworthy that the modifications of C2 or triphenylphosphonium are located adjacent to the FTY720 hydroxyl group that becomes phosphorylated on FTY720, suggesting that our modifications likely prevented FTY720-C2 or FTY720-Mitoxy from accommodating interaction with the active site of sphingosine kinase 2, the main FTY720 phosphorylating kinase [46]. Overall, the in vitro metabolism of FTY720-C2 and FTY720-Mitoxy appeared to be quite similar to that of the parent FTY720, except for the fact that no phosphorylated metabolites were detected for either new compound.

Following oral or IV administration to mice, FTY720-C2 levels were detected in both plasma and brain up to 8 hr postdose (Fig 5A and 5B), but plasma and brain concentrations dropped to unmeasurable levels at 24 and 48 hr postdose after either route of delivery. In contrast, FTY720-Mitoxy delivered by IV dosing was detected both in plasma and brain up to 48 hr postdose (Fig 5C), our last sampling time point. However, no FTY720-Mitoxy was ever detected in plasma or brain after oral delivery, indicating a lack of oral bioavailability for this compound.

The blood-brain-barrier is a major obstacle for the penetration of many compounds into brain [47]. Given that the pharmacologically active site for both new compounds is expected to be brain, the ability of both FTY720-C2 and FTY720-Mitoxy to penetrate the blood-brain-barrier, within 5 min of delivery (Fig 5A and 5C), is a very important and desirable property for therapeutics. Also, in general, there was a time-dependent increase of both new compounds in the brain-to-plasma ratios. Notably, the elimination half-life of FTY720-Mitoxy was approximately two times longer in brain (1.77 hr) than in plasma (0.94 hr). These data suggest that both analogues preferentially partition to the brain.

Due to slow absorption and high binding to plasma proteins [48], FTY720 half-life in rat was found to be ~20 hr [49] with single IV doses ranging from 0.3 to 4 mg/kg, and at ~7 days in human after a single oral doses of 1 mg FTY720 [50]. In comparison to FTY720, neither of the new analogues was phosphorylated and had markedly shorter half-lives (less than 2 hr, for FTY720-Mitoxy) than the parent compound. Taking into account that FTY720-C2 and FTY720-Mitoxy showed species-dependent differences in metabolism; if their pharmacokinetic data in mice translates to humans, this would likely have an impact on dosing regimens for the new compounds. With the ability to rapidly enter brain, optimal levels might be accomplished using novel sustained-release-drug-delivery systems.

It is interesting to note, that PP2A activity was significantly increased in mouse adrenal gland after single oral or IV doses of FTY720-C2 (1 mg/kg) or a single IV dose of FY720-Mitoxy (2 mg/kg), even though there was no phosphorylation of FTY720-C2 or FTY720-Mitoxy noted. This might have been anticipated considering that dephosphorylated, not phosphorylated FTY720, efficiently increases PP2A activity in vitro [17, 28]. The PP2A stimulatory effect was observed at all sampling time points (Fig 6), including 24 hr and 48 hr postdose for FTY720-C2, when drug was no longer detectable in blood or brain. This may be due, at least in part, to binding properties of these compounds with lipids, which are abundant in the adrenal medulla [51]. Furthermore, αSyn positively regulates PP2A under normal physiological conditions, especially when αSyn is dephosphorylated [14, 16], and Lewy-body-like αSyn aggregation causes loss of PP2A activity in adrenals of aging synucleinopathy mice as well as in brain from patients with Dementia with Lewy bodies (DLB) or PD [15, 20].

PP2A is a major serine/threonine phosphatase in the brain that regulates the phosphorylation state of many neuronal proteins, including two key enzymes involved in dopamine biosynthesis, tyrosine hydroxylase and aromatic amino acid decarboxylase [13, 14, 52]. One in vivo study that enhanced PP2A activity, demonstrated improved neuronal activity, reduced glial activation, and better motor performance in a mouse model that develops αSyn aggregates in the brain [21]. Therefore, our data suggest that FTY720-C2 and FTY720-Mitoxy may help promote or reverse impaired physiology by restoring dysregulated PP2A activity due to synucleinopathy in PD, DLB, and multiple system atrophy (MSA). Moreover, PP2A activity is decreased in AD brain [53] in which PP2A normally negatively regulates phosphorylation of the microtubule-associated protein tau [53, 54], which is strongly implicated in AD associated neurofibrillary tangle formation due to tau hyperphosphorylation [55, 56]. These findings raise the potential importance of therapies that can sustain optimal PP2A activity. Other studies using PP2A-activity stimulators also show reduced abnormal tau phosphorylation and improved spatial learning and memory in aging mice and in AD transgenic mouse models [57, 58].

Like all drugs, our new FTY720-based compounds could have side effects similar to those seen with FTY720. The most commonly reported adverse events in patients taking FTY720 include bradycardia (slow heart rate), upper respiratory tract infections (primarily nasopharyngitis), sometimes serious infections, and clinically asymptomatic lymphopenia (low level of blood lymphocytes) [59]. Bradycardia can occur at the first dose but in individuals who continue taking the drug, heart rate eventually returns to baseline. Infection and lymphopenia are known to be associated with FTY720’s immunosuppressive effects due to its effects on T cells, as described above. FTY720 is an FDA-approved drug for multiple sclerosis (MS), a disease that is considered to be an autoimmune disorder resulting from auto-reactive immune cells attacking the myelin of the central nervous system [60]. Phosphorylated FTY720 (FTY720-P) acts as an agonist of the sphingosine 1-phosphate receptor 1 (S1P_1_), which is abundant on lymphocytes, and consequently causes S1P_1_ internalization that prevents lymphocyte egress from peripheral lymph nodes [61] and subsequent entry into the circulation [62–64]. Unlike FTY720, neither FTY720-C2 nor FTY720-Mitoxy undergo metabolic phosphorylation (Figs 1 and 3), which suggests that they will not act on S1P_1_, and therefore should not cause immunosuppressive side effects, which is currently being investigated in mice. Overall, both new FTY720-based compounds, with brain penetration, may be quite suitable and/or beneficial for treating neurodegenerative diseases with dysregulated PP2A activity and no need for immunomodulation.

While this preliminary study had a sample size of two (n = 2) mice per drug and time point (N = 36 total), it has allowed us to characterize key pharmacokinetic aspects of our new compounds. FTY720-C2 is orally bioavailable and both FTY720-C2 and FTY720-Mitoxy rapidly cross the blood-brain-barrier, preferentially partition to brain, and modulate PP2A activity in vivo. Also, FTY720-C2 and FTY720-Mitoxy had shorter half-lives than FTY720, suggesting higher clearance, which may also be beneficial. While we acknowledge that variability of a population from which a sample is drawn can be over- or underestimated when sample size is small, it is important to note that statistical analysis with two values is considered valid based on mathematical equations, and as also validated using computer simulations [65]. In conclusion, further characterization as well as safety assessment of both FTY720-C2 and FTY720-Mitoxy will be important preclinical assessments to perform prior to initiating clinical trials with these novel compounds.
